# Questionnaire-Derived Sleep Habits and Academic Achievement in First Year University Students

**DOI:** 10.3390/clockssleep4010001

**Published:** 2021-12-28

**Authors:** Matthew Driller, Haresh Suppiah, Paul B. Gastin, Christopher M. Beaven

**Affiliations:** 1 Sport and Exercise Science, School of Allied Health, Human Services and Sport, La Trobe University, Melbourne 3083, Australia; h.suppiah@latrobe.edu.au (H.S.); p.gastin@latrobe.edu.au (P.B.G.); 2 Te Huataki Waiora School of Health, University of Waikato, Tauranga 3116, New Zealand; martyn.beaven@waikato.ac.nz

**Keywords:** pittsburgh sleep quality index, student success, grades, sleep hygiene

## Abstract

This study aimed to determine the effect of sleep quantity and quality via the Pittsburgh Sleep Quality Index (PSQI) on students’ academic achievement in their first year of university study. In this cross-sectional study, 193 students (102 female, 91 male, mean ± SD; age = 19.3 ± 2.9 y) from an undergraduate Health degree in New Zealand completed the PSQI four weeks prior to the end of the semester in their first year of university study. Results from three core subjects in the first semester were averaged and correlations between the PSQI and academic success were evaluated using Spearman’s rho (ρ). The group were also trichotomized using a PSQI global score of ≤5 as the threshold for “good” sleepers (*n* = 62, 32%), a score of 5–8 for “moderate” sleepers (*n* = 63, 33%) and a score ≥8 to characterize “poor” sleepers (*n* = 68, 35%). Overall, students averaged 7 h 37 min of self-reported sleep duration with an average bedtime of 22:55 p.m. and wake time of 8:01 a.m. There was a significant, small inverse relationship between academic performance and bedtime (*p* = 0.03, ρ = −0.14), with those going to bed earlier having superior academic success. The trichotomized data demonstrated no significant differences in academic performance between students with poor, moderate and good sleep quality (*p* = 0.92). Later bedtimes were associated with lower academic performance in a group of first year university students. However, there were no other relationships observed between academic success and self-reported sleep quality or quantity as determined by the PSQI. Enhancing awareness of the impact of sleep timing on academic success should be prioritized and strategies to improve sleep hygiene should be promoted to university students.

## 1. Introduction

The first year of university study often coincides with students leaving home and becoming more responsible for their own health and well-being behaviours, including their sleep habits and routines [1]. In the lead-up to university, through adolescence, there is a progressive delaying of the chronotype until approximately 20 years of age [2]. This sleep-phase shift is typically attributed to psychosocial factors and to developmental changes in the brain that take place during puberty [3,4], and the peak in this shift often coincides with the first year of university study. University students are well known for their erratic sleep schedules and late bedtimes [5,6], with up to 50% of students reporting significant levels of daytime sleepiness [7]. In this regard, university students are particularly at risk of developing sleep disorders, with previous research also suggesting negative effects on their academic performance [8,9], often related to impaired concentration following poor sleep [10].

In 2014, a study of ~1300 New Zealand university students aged 16 to 38 years investigating measures of mental health and well-being highlighted substantial health concerns [11]. In this cohort, 39% were suffering from significant sleep disorders, with depression, anxiety, illicit substance use, and circadian rhythm disorders being the most common causes of symptoms. Furthermore, previous work from our laboratory evaluating health, fitness, and body mass changes in first year university students showed that 50% of participants reported declining self-reported sleep practices across a 12-week university semester [1].

Concurrently, previous research has suggested negative effects of poor sleep indices on academic performance [8,12,13]. Gaultney [8] sampled 1845 university students in America with a validated sleep disorder questionnaire. Results showed that 27% of students were at risk for at least one sleep disorder. Furthermore, students at risk for sleep disorders were overrepresented among students in academic jeopardy, with a grade point average (GPA) of <2.0. Similarly, Gomes et al. [14] surveyed 1654 full-time undergraduate students in Portugal using a questionnaire on sleep, academics, lifestyle and well-being [14]. The main predictors of academic success in their study were: self-reported sleep quality and self-reported frequency of sufficient sleep, class attendance, night outings and previous academic achievement [14].

The impact of sleep loss on academic performance might be subtle. In a study by Engle-Friedman et al. (2003), 50 university students were deprived of sleep for one night, and were then asked to solve math addition problems [15]. Participants self-selected the difficulty level of the problems and after the sleep loss condition, participants were more likely to choose easier problems to solve. From these data, authors suggested that students who are chronically sleep deprived may limit their future options by choosing easier courses while in university. Supporting this theory, Okano et al. (2019) issued 88 wearable sleep monitors to a university chemistry class at Massachusetts Institute of Technology for a semester of study, allowing for multiple sleep measures to be correlated with academic performance [16]. Overall, better quality, longer duration, and greater consistency of sleep correlated with better grades. However, there was no relationship between sleep measures on the single night before a test and test performance; instead, sleep duration and quality for the month and the week before a test correlated with better grades [16].

Not all studies have reported the same relationship between sleep duration and academic performance. Eliasson (2010) suggested that bed times, sleep latency, and wake times had a greater impact on academic performance than sleep duration in 157 American university students [13]. Sweileh and colleagues (2011) also reported no relationship between sleep quality via the Pittsburgh Sleep Quality Index (PSQI) and academic success (via a self-reported 4-point scale) in 400 Palestinian undergraduate students [17]. In another study on 189 medical students in Pakistan, there was no significant association between lack of sleep and test scores [18]. The inconsistencies in both measuring sleep (e.g., subjective or objective tools), reporting of academic performance (grades or self-reporting of success), as well as the different cultural, year-level and field of study contexts where the research has taken place, make it difficult to draw any definitive conclusions on the relationship between sleep and academic performance.

While numerous studies have indicated that sleep issues may be an area for concern regarding overall wellbeing and academic success in university students, there are yet to be any studies that have evaluated the potential link between sleep and academic performance in the New Zealand university context. Furthermore, insights into this link between sleep and academic performance amongst students studying in a health-related field, where you could suggest the students may be better informed, also warrant further investigation. Given the inconsistencies in the literature, in addition to the lack of information on sleep and academic performance in the uniquely multi-cultural New Zealand university setting, our study sought to further identify this relationship. Therefore, the aim of the current study was to determine the effect of sleep quantity and quality on students’ academic performance in their first year of university study in New Zealand, where previous research has suggested that university students are at risk of sleep disorders [1,11]. The current study opted for the use of a validated sleep questionnaire (PSQI) in combination with academic grades attained from the registrar during the first year of university study, with the expectation that positive sleep duration and quality metrics from the PSQI would be related to an overall increase in academic performance.

## 2. Results

There were no significant differences (*p* = 0.32) between male and female students for PSQI global scores (6.7 ± 3.1 and 6.8 ± 2.7, respectively). A total of 68 (35%) participants were identified as being poor sleepers, 63 (33%) as moderate sleepers and the remaining 62 participants (32%) were classified as good sleepers. Overall, self-reported mean bedtime was 22:55 p.m. ± 1:20 h:min and wake time was 08:01 a.m. ± 1:27 h:min with an average sleep duration of 7:37 ± 1:15 h:min. Mann–Whitney U-tests revealed no significant differences in academic results, bedtime and waketime between participants with poor, moderate, and good sleep quality. However, significant differences existed in sleep efficiency, total sleep time and PSQI global score (*p* < 0.001). The detailed differences between poor, moderate, and good sleep quality are presented in Table 1.

Table 2 summarizes the strength of relationships between academic grade/result and sleep characteristics. Academic results had a small inverse association with bedtime (*p* < 0.05). Waketime and sleep efficiency were associated with bedtime (*p* < 0.001). There was a significant inverse association between total sleep time and bedtime (*p* < 0.01). Total sleep time (*p* < 0.001), sleep efficiency (*p* < 0.001) and PSQI global scores (*p* < 0.05) were associated with waketime. Sleep efficiency was associated with total sleep time (*p* < 0.001). PSQI global score was inversely associated with total sleep time (*p* < 0.001). Finally, PSQI global score was negatively associated with sleep efficiency (*p* < 0.001).

## 3. Discussion

The main findings from the current study showed that there was a significant inverse relationship between average grades and bedtime, with students who went to bed earlier having higher academic grades during the first semester of university study. However, there were no other relationships observed between academic success and self-reported sleep quality or quantity as determined by the PSQI. Poor sleep was common in this studied cohort, with 35% of students classified as poor sleepers based on their PSQI score > 8. Over the entire cohort, mean sleep duration was 7 h 27 min, with mean bed and wake times of 22:55 p.m. and 8:01 a.m., respectively.

Sweileh et.al. (2011) also reported no relationship between sleep quality and academic success in Palestinian undergraduate students [17]. In their study, they demonstrated that complaints about sleep problems were common among university students, with approximately 28% of students evaluating their sleep quality as ‘satisfactory’ or ‘poor’ on a four-point scale. In comparison, the current study found that 68% of students had moderate to poor sleep quality as assessed by the PSQI. The lack of findings for the relationship between academic grades and TST has also been reported previously. Eliasson (2010) reported that sleep factors such as bed and wake times had a greater impact on academic performance than TST in American university students [13]. Similar to the current study, when compared to lower-performing students, higher-performing students had an earlier sleep onset time with no significant difference in TST.

Our results contrasted with those reported by Gaultney et al. (2010), who reported that students who were at risk for sleep disorders (27% of their sampled population) were overrepresented among students in academic jeopardy amongst 1845 university students in America. While sleep disorders were not specifically measured in the current study, PSQI global scores >6 have been used previously to indicate that individuals may be suffering from sleep disturbances, such as insomnia [19]. However, when stratified into three groups by their PSQI scores (good, moderate and poor sleepers), there were no differences in academic scores in the current study. A potential reason for these differences in comparison to the Gaultney et al. study might be due to the overall differences in sleep habits. We reported a mean sleep duration of 7 h 37 min across our student population, while Gaultney et al. reported a mean sleep duration of ~6 h 47 min during the school week. It is possible, therefore, that the differences in sleep duration of almost one hour per night may have also translated to poorer sleep behaviours in the American students. While it is difficult to make further comparisons between the study cohorts, the Gaultney et.al. study was performed on Psychology students, while the current study was on Health, Sport and Human Performance students. There may also be cultural differences that exist around sleep habits and behaviours between students in America and students in New Zealand.

We acknowledge that the current study targeted a very specific subset of a New Zealand university population. While the ethnicity break-down of our sample population was representative of the entire university population, this population of students were all studying their first semester of a Bachelor’s degree in Health, Sport and Human Performance. Given the topic areas of study in this semester included health and wellbeing, human performance, nutrition and physical activity, we could assume that these students may have additional knowledge and a vested interest in maintaining a healthy lifestyle, including their sleep behaviours. Furthermore, the average sleep duration in the current study (7 h 37 min) was higher than that reported in university students previously [6,14,20]. In a study of 19,417 university students across 26 countries, mean self-reported sleep duration was ~7 h, considerably less than the current study. However, it appears that even students studying in health-related fields are not immune to the reported declines in health and well-being during this stage of life [21,22].

This study is not without its limitations. While comparable to previous research in other countries and university settings [13,15,18], the sample size in the current study was relatively small (*n* = 193). However, this was largely due to the size of the overall population to draw from studying in the same degree program. Indeed, our population represented 87% of the whole cohort and was extended over a two-year period to increase the sample size. The authors considered extending the research to students undertaking other fields of study within the same university; however, we felt that a novel aspect was to examine the relationship between academic success and sleep in a group of students who would have been more aware of the potential impact of sleep on overall health and performance. Future research should aim to evaluate whether the findings in the current study are similar to those in students across other fields of study in non-health-related programs (e.g., Computer Sciences, Engineering, Law). Furthermore, investigating other factors that may impact on sleep habits in the university student population, for example, their living environments (e.g., on-campus in university accommodation, off-campus or at home with parents) and also their chronotype (e.g., morning or evening types) may yield other interesting insights that warrant further research. Lastly, the use of self-reported sleep information via the PSQI, as opposed to objective data (e.g., actigraphy), could be considered a limitation of the current study.

The practical implications for the findings in the current study may include the need for greater flexibility in the timing of course offerings at university. Students in the current study showed a range of reported bed and wake times, suggesting that many chronotype differences exist across the cohort. Flexibility of class schedules, as opposed to rigid schedule requirements, may allow students to opt for a more preferred class timetable, allowing for an individualized plan based on sleep–wake cycles and potentially, improved academic performance [23].

In the current study, later bedtimes were associated with lower academic grades in a group of 193 first year university students in New Zealand. However, there were no other relationships observed between academic success and self-reported sleep quality or quantity as determined by the PSQI, nor were there any differences when students were stratified into three groups of good, moderate and poor sleep quality. Interestingly, total sleep duration did not correlate with grades, suggesting that timing of sleep onset may be a more important contributor to academic performance; however, further research is warranted across larger cohorts to confirm these findings. Overall, the mean reported sleep duration in the current study was over 7.5 h, which is somewhat higher than previous studies on university students [20]; this may reflect the field of study in these students (Health, Sport and Human Performance), with a higher level of assumed knowledge on the importance of sleep to general health and wellbeing.

## 4. Materials and Methods

A convenience sample of first year students (*n* = 193) over two consecutive years at a New Zealand university volunteered to participate in the study (102 female, 91 male, mean ± SD; age = 19.3 ± 2.9 y). Participants were ethnically diverse (61% NZ European, 21% Māori, 11% Pacific Islander, and 7% classified as “other ethnicity”). All students were part of the same faculty, studying towards a Bachelor’s degree in Health, Sport and Human Performance. Students that were studying full-time were invited to participate via email. The 193 students that agreed to participate in the study were a representative sample, accounting for 87% of the total enrolment in the course at first-year level. Ethical approval for the study was granted by the institution’s Human Research Ethics committee (HREC2018#63) and all participants provided written informed consent.

The data collection instruments were a demographic form including age, sex and ethnicity, and the Pittsburgh Sleep Quality Index (PSQI). These forms were completed via an electronic online survey (Survey Monkey, Palo Alto Inc., Santa Clara, CA, USA). The PSQI is a self-report questionnaire that examines the quality of sleep and sleep disturbance over a 1-month period in clinical and nonclinical populations [24]. The PSQI has been demonstrated to have good internal reliability, validity and is accepted as the most commonly-used subjective sleep measure, not only in the research literature, but also in the sleep community [24].

The PSQI evaluates sleep quality and sleep disturbance during the previous one-month period. The scale begins with four open-ended questions: “When have you usually gone to bed at night?”, “How long has it taken you to fall asleep each night?”, “When have you usually gotten up in the morning?”, and “How many hours of actual sleep did you get at night?”. Following these are 14 questions rated on 4-point scales, where lower scores indicate better sleep quality. The 18 items are used to create seven components: subjective sleep quality, sleep latency, sleep duration, habitual sleep efficiency, sleep disturbances, use of sleeping medication, and daytime dysfunction. Each component score has a range of 0 = No difficulty to 3 = Severe difficulty, and each is derived from one or more of the qualitative or quantitative scale items. The seven component scores are summed, making up the total/global score ranging from 0 to 21, with higher scores representing a lower sleep quality [24]. In analysing the data, PSQI scores were trichotomized into three groups. A PSQI score of ≤5 was considered the threshold for “good” sleep quality, a score of ≥8 was characterized as “poor” sleep quality, while those between 5 and 8 were characterized as having “moderate” sleep quality [25].

Academic grades across three core subjects in the first year of the Bachelor’s degree in Health, Sport and Human Performance were obtained for all participants. These subjects included: Introduction to Human Performance Science, Foundations of Health, Sport and Human Performance, and Understanding Health and Wellbeing. The final overall grade (out of 100) at the end of the semester for each subject was used for analysis, and the average for each individual across these three subjects was used to represent their overall academic performance.

### Statistical Analysis

Statistical analysis was conducted using R (Version 4.0.2, R Core Development Team). The level of statistical significance for all analyses was set at *p* < 0.05. Descriptive data were expressed in means and standard deviations with data normality assessed through Shapiro–Wilk normality tests. As data were not normally distributed (*p* < 0.05), statistical comparisons were conducted using non-parametric statistics. A Mann–Whitney U test was initially run to determine if there were differences in sleep quality (PSQI global scores) between males and females. As no differences in sleep quality were found between sexes (*p* = 0.32), data were pooled for analysis. A Kruskal–Wallis test was conducted to examine differences by sleep quality type on the pooled data. Effect sizes were calculated using epsilon square (ε^2^) [26], and interpreted as follows: 0.00 < 0.01—negligible; 0.01 < 0.04—weak; 0.04 < 0.16—moderate; 0.16 < 0.36—relatively strong; 0.36 < 0.64—strong; 0.64 < 1.00—very strong [27]. This was followed by the Dwass–Steel–Critchlow–Fligner test for multiple comparisons. Spearman’s rho (ρ) was used to quantify associations between grade, bedtime, waketime, total sleep time, sleep efficiency, and PSQI global score.

## Figures and Tables

**Table 1:** Comparison between students with good (≤5 in PSQI) moderate (6–7 in PSQI) and poor (≥8 in PSQI) sleep quality. Data shown as means (SD).

Measure	Good (*n* = 62)	Moderate (*n* = 63)	Poor (*n* = 68)	Overall			Good vs. Moderate	Good vs. Poor	Moderate vs. Poor
				χ^2^	*p*-Value	Effect Size (ε^2^)	*p*-Value	*p*-Value	*p*-Value
Grade	77.6 (11.2)	76.9 (11.1)	76.9 (11.8)	0.100	0.951	0.001	0.958	0.967	0.992
Sleep efficiency (%)	92.9 (7.6)	85.2 (9.2)	77.5 (17.0)	43.607	<0.001	0.227	<0.001	<0.001	0.003
Total sleep time (h:min)	8:11 (0:59)	7:46 (1:06)	7:00 (1:21)	33.388	<0.001	0.174	0.154	<0.001	<0.001
PSQI Global	3.6 (1.2)	6.4 (0.5)	10.0 (1.8)	173.161	<0.001	0.902	<0.001	<0.001	<0.001
Bedtime (p.m.)	23:00 (01:27)	22:41 (01:04)	22:58 (01:23)	2.207	0.332	0.011	0.432	0.996	0.374
Waketime (a.m.)	07:51 (01:29)	07:52 (01:12)	08:17 (01:39)	5.788	0.055	0.030	0.706	0.069	0.184

**Table 2:** Spearman correlation matrix between academic grade results and sleep variables, including PSQI global score. * *p* < 0.05, ** *p* < 0.01, *** *p* < 0.001. TST, Total sleep time; PSQI, Pittsburgh Sleep Quality Index.

Measure	Academic Results		Bedtime		Waketime		TST		Sleep Efficiency		PSQI	
Academic Results	-											
Bedtime	−0.16	*	-									
Waketime	0.01		0.27	***	-							
TST	0.09		−0.23	**	0.26	***	-					
Sleep Efficiency	−0.10		0.34	***	−0.35	***	0.39	***	-			
PSQI	−0.03		−0.01		0.16	*	−0.47	***	−0.52	***	-	
